# Supplementary figures and images for: Refined Quantification of Infection Bottlenecks and Pathogen Dissemination with STAMPR
Source: mSystems. 2021 Aug 17;6(4):e00887-21. doi: 10.1128/mSystems.00887-21 (PMC8407386; doi:10.1128/mSystems.00887-21)

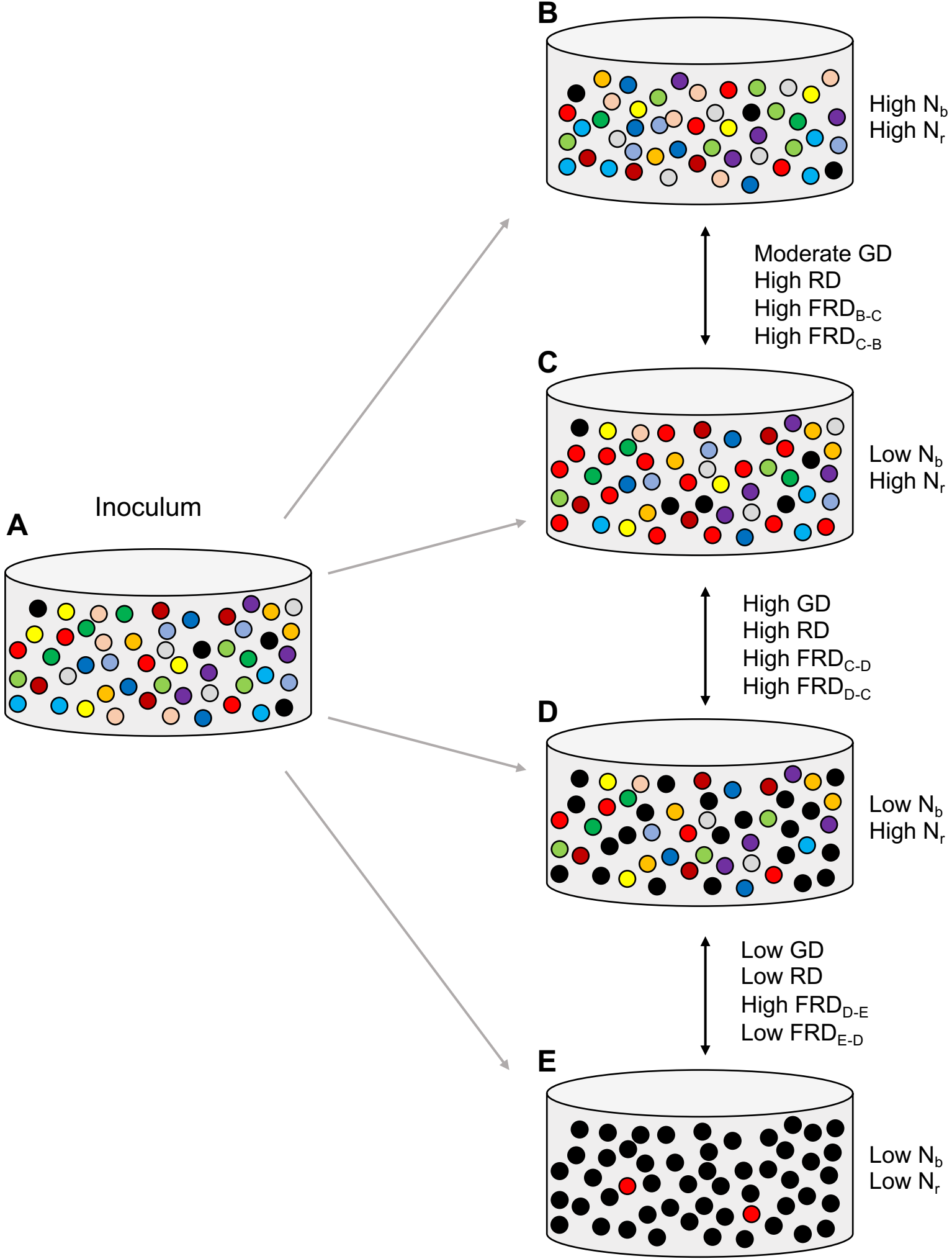

Supplement: FIG S1 [file msystems.00887-21-sf001.pdf]

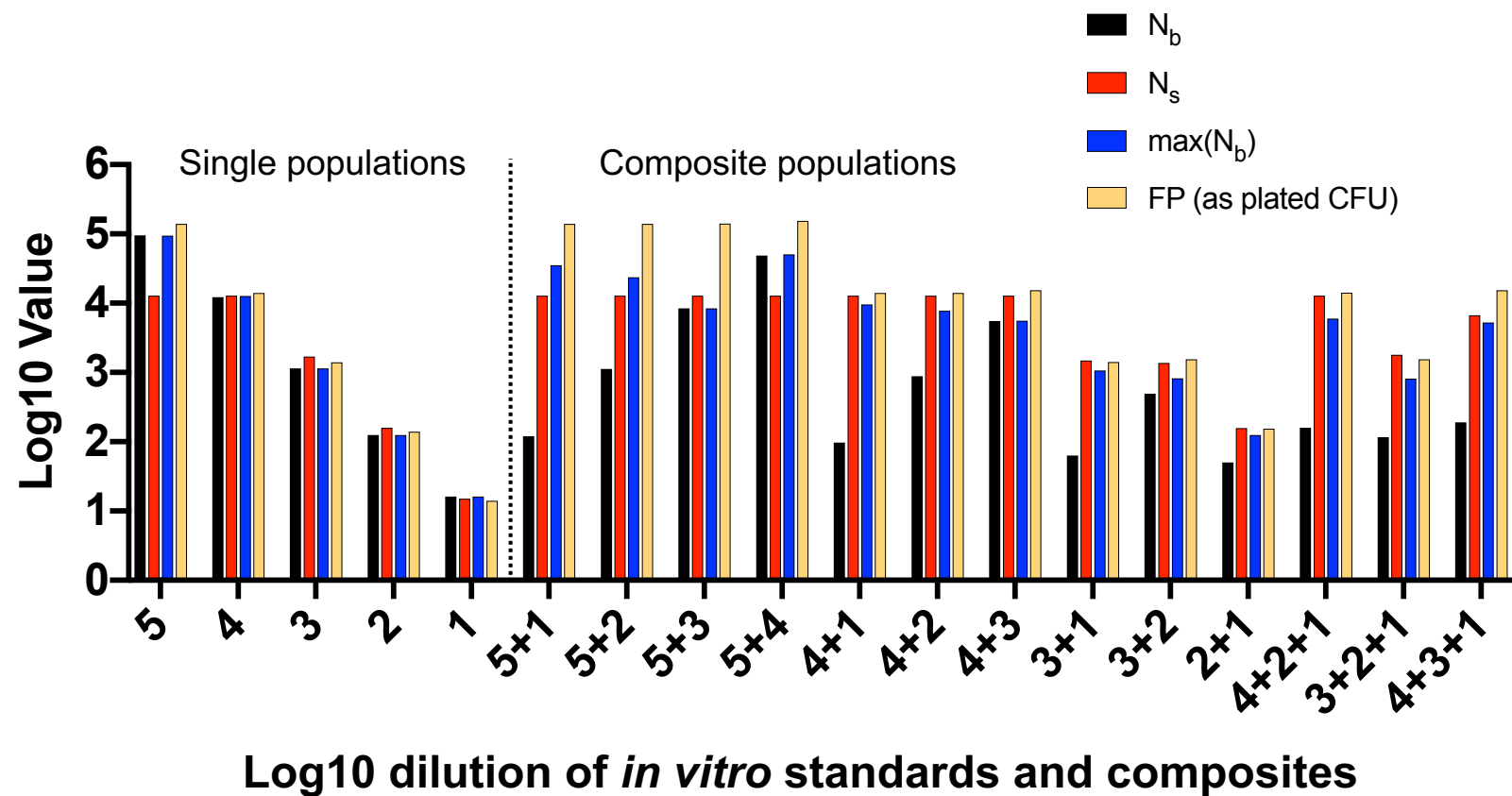

Supplement: FIG S2 [file msystems.00887-21-sf002.pdf]

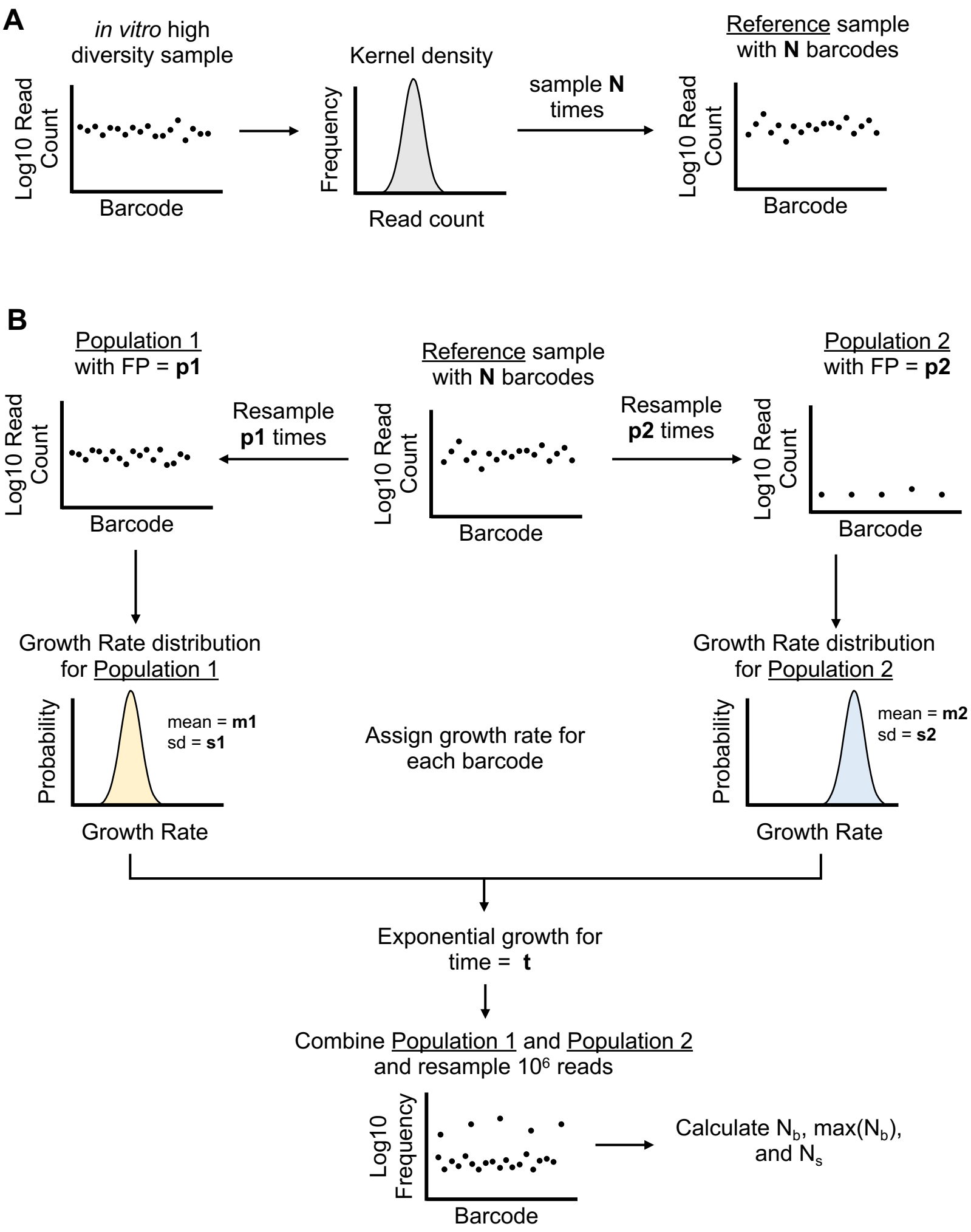

Supplement: FIG S3 [file msystems.00887-21-sf003.pdf]

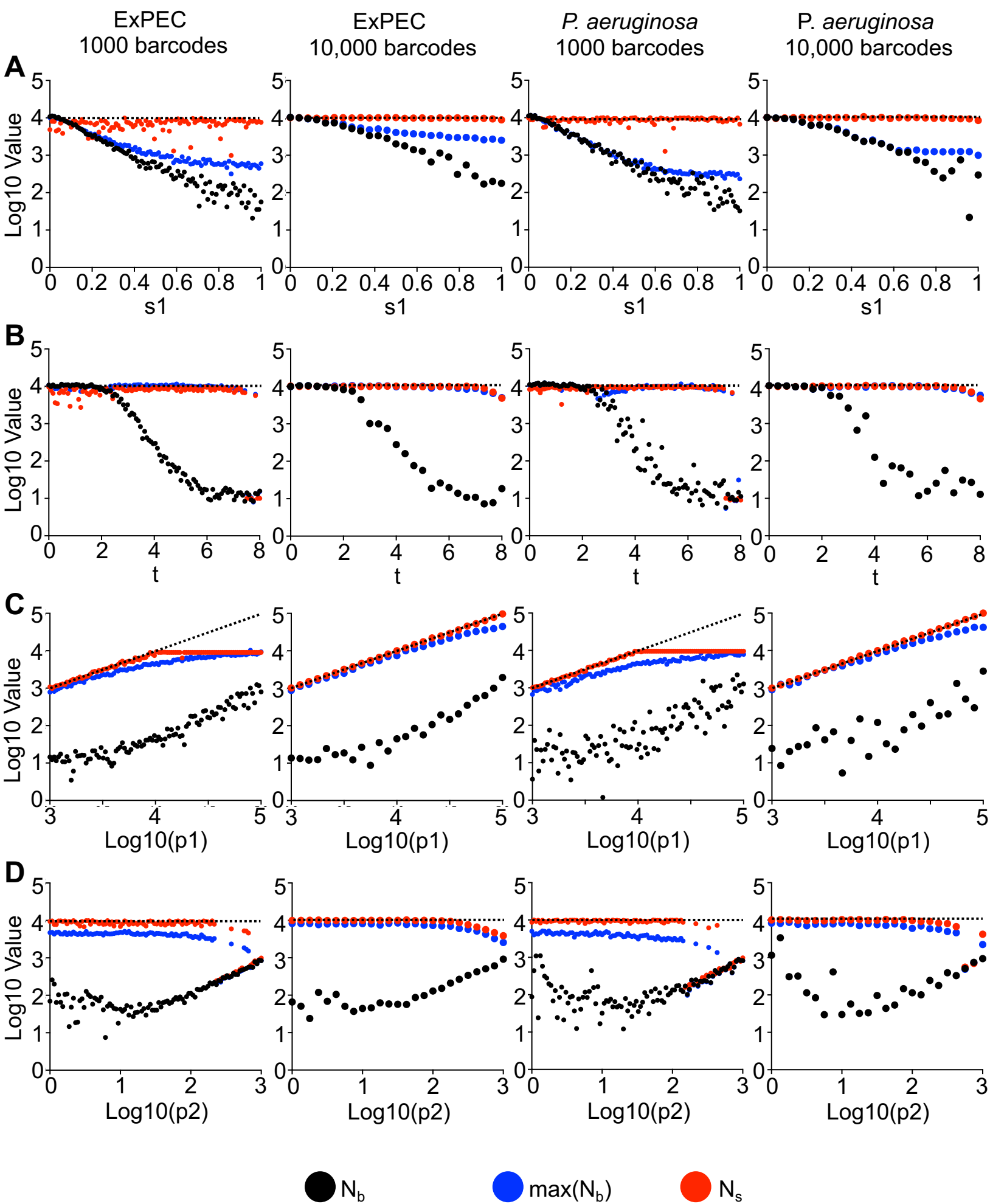

Supplement: FIG S4 [file msystems.00887-21-sf004.pdf]

**A**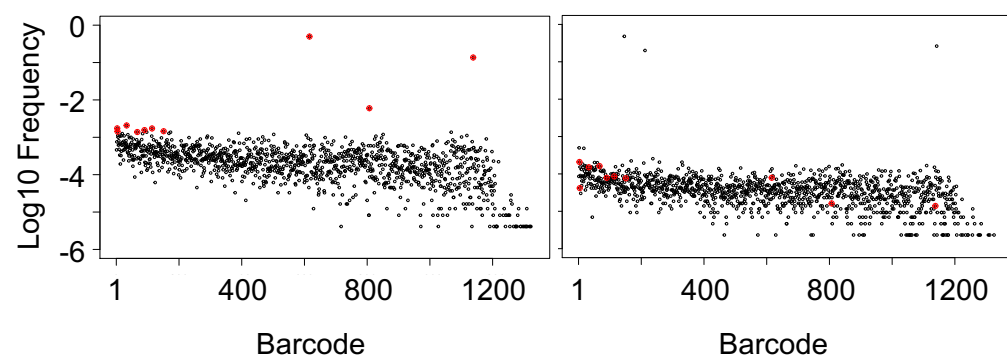**RD = 1183   GD = 0.822**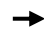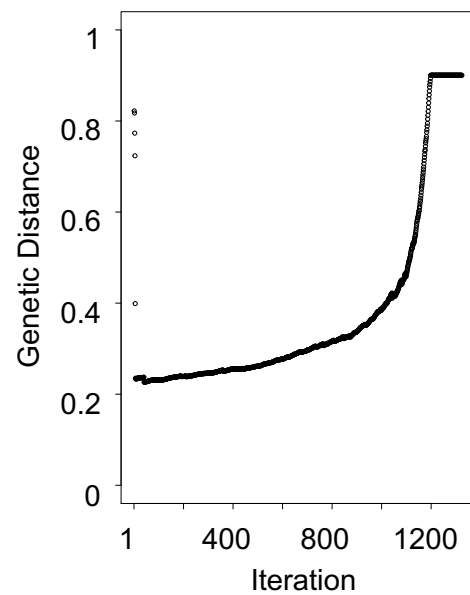**B**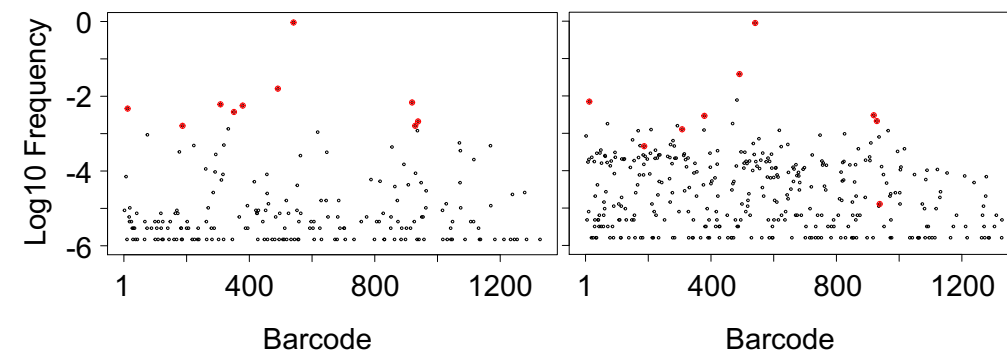**RD = 7   GD = 0.016**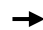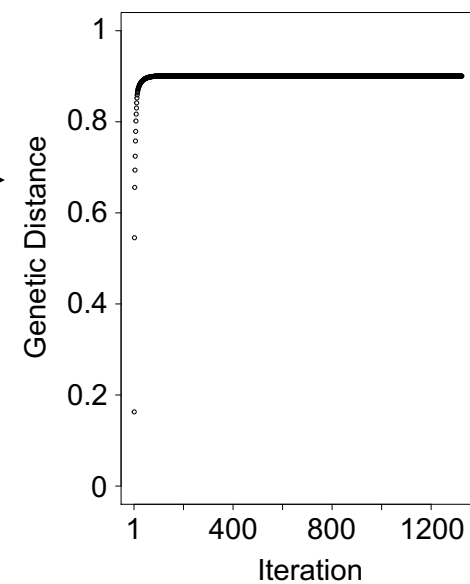**C**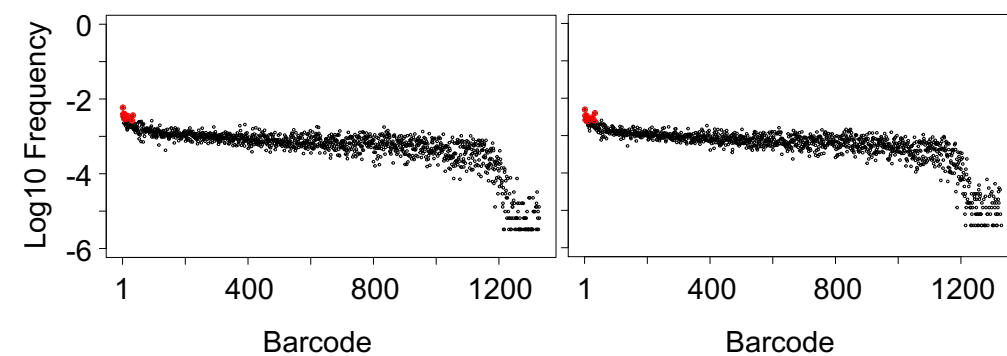**RD = 1283   GD = 0.051**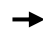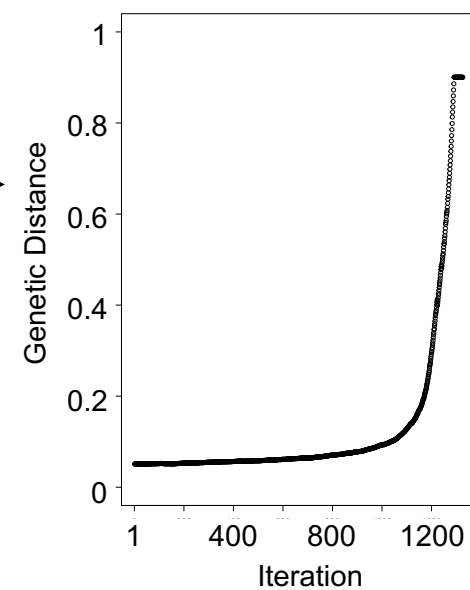

Supplement: FIG S5 [file msystems.00887-21-sf005.pdf]

### Mouse 2

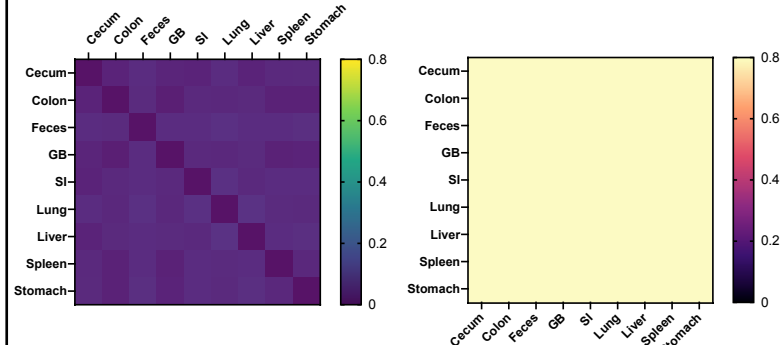

### Mouse 6

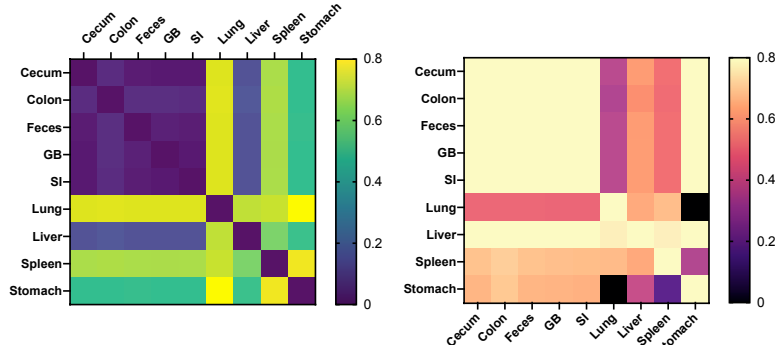

### Mouse 3

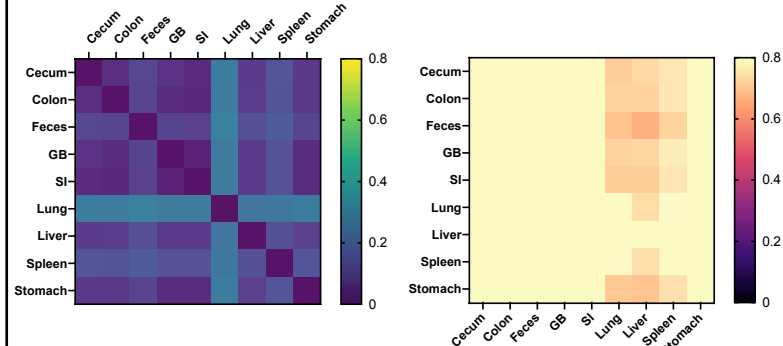

### Mouse 7

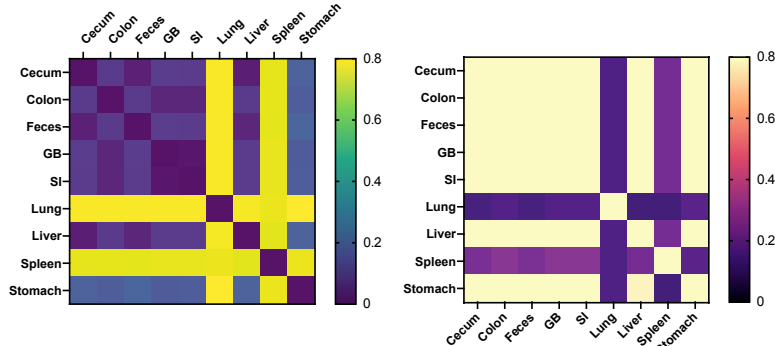

### Mouse 4

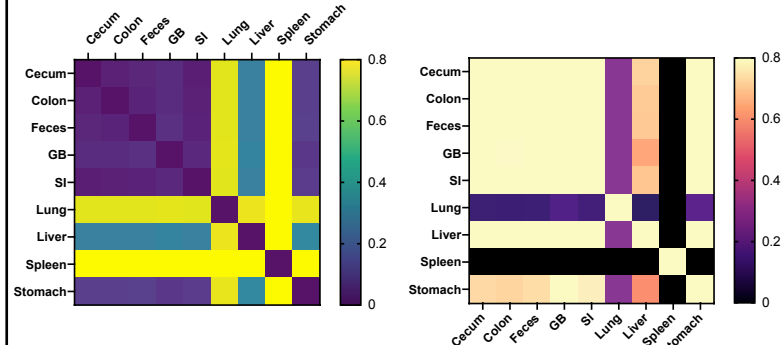

### Mouse 8

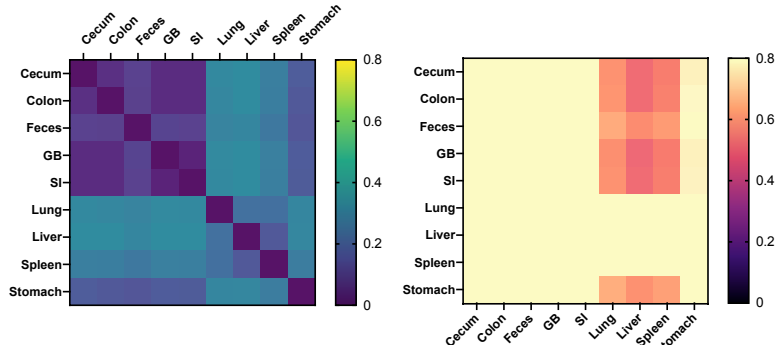

### Mouse 5

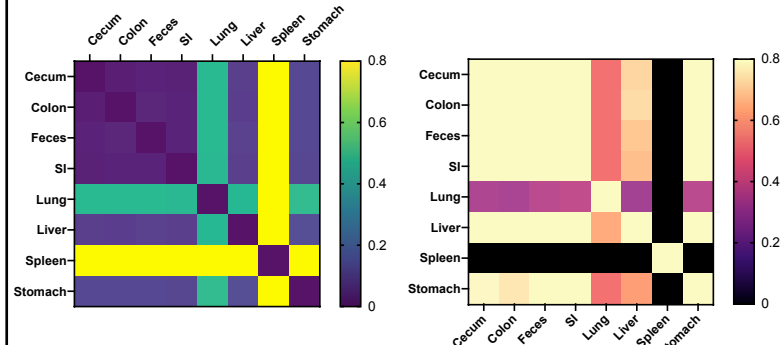

### Mouse 9

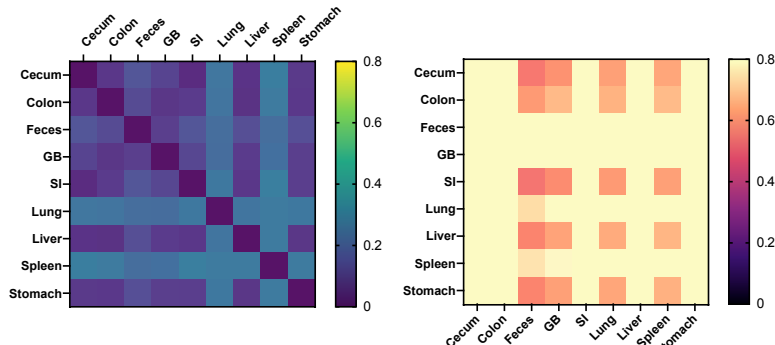

### Mouse 10

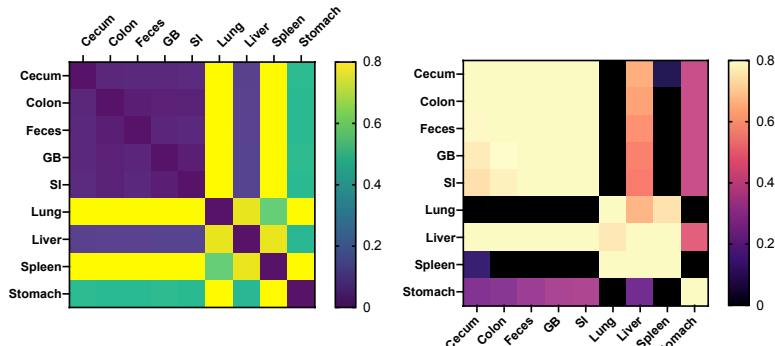

Supplement: FIG S6 [file msystems.00887-21-sf006.pdf]

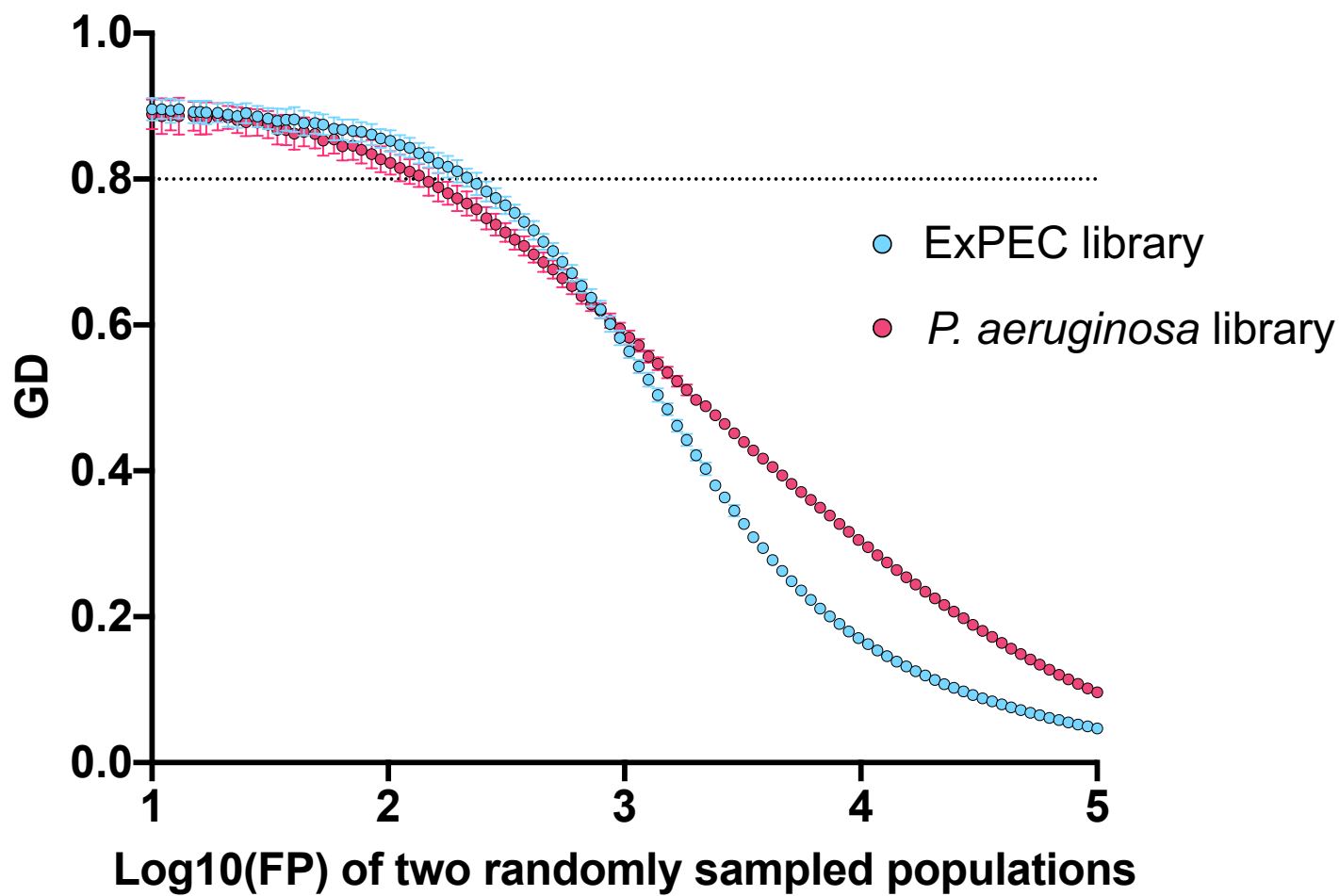

Supplement: FIG S7 [file msystems.00887-21-sf007.pdf]
